# Screening Children in India: Translation and Psychometric Evaluation of the Parents’ Evaluation of Developmental Status and the Strength and Difficulties Questionnaire

**DOI:** 10.3390/pediatric15040067

**Published:** 2023-12-14

**Authors:** Hina Sheel, Lidia Suárez, Nigel V. Marsh

**Affiliations:** 1 School of Social and Health Sciences, James Cook University, Singapore 387380, Singaporelidia.suarez@jcu.edu.au (L.S.); 2 School of Health and Life Sciences, De Montfort University, Academic City, Dubai 294345, United Arab Emirates

**Keywords:** PEDS, SDQ, translation, cross-validation, children, India

## Abstract

Timely screening and surveillance of children for developmental delay and social–emotional learning difficulties are essential in Low- and Middle-Income Countries like India. Screening measures like the Parents’ Evaluation of Developmental Status (PEDS) and Strength and Difficulties Questionnaire (SDQ) are considered suitable for India due to their low cost, easy accessibility, and no training requirement for administration. However, India lacks validated screening measures, and the PEDS and SDQ have yet to be validated for children in India. The study aimed to translate the PEDS and SDQ from English to Hindi and psychometrically evaluate the same measures on children aged 4–8 years in India. The original PEDS and SDQ forms and their translations were pilot tested on 55 participants and evaluated using data from 407 children with typical development (TD) and 59 children with developmental disability (DD). Parents and teachers reported no meaningful discrepancy between the original and translated (Hindi) questionnaires. Internal consistency for the PEDS was acceptable, but unacceptable for most subscales on the SDQ, for both TD and DD samples. Test–retest reliability was poor for the PEDS but adequate for the SDQ. Results from known-group validity testing showed that the PEDS scores could be used to distinguish between the TD and DD samples. The results from this study provide further support for the use of the PEDS and SDQ in developing countries like India.

## 1. Introduction

Meeting developmental milestones is a crucial aspect for the growth of a healthy child [1]. *The Diagnostic and Statistical Manual of Mental Disorders, Fifth Edition, Text Revision (DSM-5-TR)* classified DD as an Intellectual Developmental Disorder (IDD), characterized by a lack of general mental capacities and adaptive functioning [2]. Individuals with DD may struggle with social relationships when compared to neurotypical peers, and they may read nonverbal and subtle social signs differently [3]. Evidence from global health databases suggests that about 240 million children globally have developmental disabilities, with the majority of these children being from Sub-Saharan Africa (69.9 million) and South Asian countries (64.4 million children) [4]. In India, about 10% of children have developmental delays resulting in disabilities, while nearly 6% of infants are born with congenital disabilities [5]. Social–economic factors such as poverty, poor health, lack of infrastructure, and limited developmental literacy are risk factors for the delayed identification of children with impaired developmental, cognitive, and social–emotional functioning [6]. Cities across India lack clinics and trained professionals to provide rigorous diagnoses and interventions under one roof [7]. Furthermore, there is limited research conducted on children from a specific population and a lack of comparative studies between children with TD and DD in India [7,8]. Therefore, timely screening and surveillance of children in Low- and Middle-Income Countries (LMIC) is essential [1].

Developmental screening seeks to identify impairments in specific areas by healthcare professionals/doctors using a brief questionnaire [9]. Identifying which children may have a learning disability and require early intervention is the initial step [9]. Measures developed in Western countries are frequently used when assessing children for Developmental Disability (DD) and Social–Emotional Learning (SEL) in LMIC [10]. Specifically, measures from the United States and the United Kingdom are increasingly used in cultures other than those in which they were developed [11]. In theory, culture plays a vital role in the child’s social, cognitive, and motor development [10]. However, there is no consensus regarding the most effective screening tool for detecting delay and disability in children from diverse cultural settings. There is a scarcity of validated measures available to identify children with DD and SEL in LMIC [12].

The Parents’ Evaluation of Developmental Status (PEDS) and the Strength and Difficulties Questionnaire (SDQ) are considered to be suitable for a LMIC country like India because they are self-reporting, low cost, easily accessible, and do not require any training for administration [13,14]. Glascoe [15] reported the PEDS to be accurate for children in America with adequate sensitivity, specificity, interrater and test–retest reliability. In India, the English version of the PEDS has yet to be validated. The SDQ was validated on British children and had good internal consistency and predictive validity. However, the test–rest reliability was relatively poor [16].

Parents are exclusively regarded as the primary source of obtaining developmental and behavioral information about their children, and there is extensive literature to support parent-completed screening tools [13,17]. However, recently, there has been a growing appreciation for the teachers’ contribution to the screening and diagnostic process [18]. Evidence indicates that classroom teachers can reliably rate children’s learning effectiveness. Teachers can differentiate between students at risk for anti-social behavior and typically developing students at an early age [19]. Some screening tools, like the SDQ, have equivalent forms for both parents and teachers to complete.

Sheel et al. [20] conducted a scoping review of research from 1990–2020 to assess whether the PEDS, PEDS:DM, and SDQ were widely utilized in India. The review found that only a few studies have employed these screening tools. The PEDS:DM was not used, and scant published literature existed on the use of the PEDS in India. Most of the literature consisted of text and opinion-based evidence emphasizing the lack of screening in India and the limited use of the PEDS to screen children for developmental delays. Furthermore, only two studies briefly mentioned translation of the PEDS questionnaires [12,21]. The review also found that the SDQ was administered to the Indian population and used as a screening tool to compare mental health across age groups. The SDQ was found to effectively differentiate groups of individuals concerning SEL and behavioral concerns. Since India is a diverse country with many regional languages, the studies that used the SDQ catered to various population types and translated the questionnaire to regional and national languages.

In a recent study, Mukherjee et al. [22] validated the Hindi version of the PEDS and PEDS:DM on children under two years old. The study concluded that the measures are unsuitable for developmental screening due to their sub-optimal diagnostic accuracy. In addition, for the past two years, several studies have used the SDQ on children in India but have not investigated the psychometric properties of the measure [23,24,25,26,27,28]. However, it appears that the PEDS and SDQ have yet to be used together to screen children for developmental delay and SEL in India.

Understanding language is essential for all self-report instruments, where the responders answer many questions in oral and written format [29]. Thus, translating an instrument to meet another country’s language and cultural needs is essential. Abdoola et al. [30] reported that in LMIC countries, if screening tools like PEDS are not translated and adapted, they may only sometimes be appropriate developmental surveillance tools within these contexts due to cultural and linguistic differences. Rigorous translation and cultural adaptation processes for self-report instruments have not been adopted for use in DD, Autism Spectrum Disorder, and general populations [31,32]. Specifically, English and translated Hindi versions of the PEDS and SDQ are yet to be psychometrically evaluated for use with children in India [20].

Considering the need for active developmental and SEL screening and the lack of validated measures available for LMICs like India, the present study aimed to first translate the PEDS and SDQ from English to Hindi. Second, the study evaluated the psychometric properties (reliability and validity) of the translated measures when used with parents and teachers of children in India.

## 2. Measures

### 2.1. Parents’ Evaluation of Developmental Status (PEDS)

The PEDS [33] is a surveillance and screening tool for children aged 0–8 years. The tool elicits and addresses parents’ concerns about development, behavior, and mental health. The tool comprises one form with 10 questions across 10 categories (expressive language; receptive language; and social–emotional, behavioral, fine motor, gross motor, self-help, school, cognitive, and health issues). The questions in the PEDS elicit parents’ perspectives of their child’s development and their answers are used to classify their child as high, medium, or low risk. The response options are “yes”, “no”, and “a little”. The scoring for the PEDS includes columns for each age range and identifies which concerns predict problems and which do not. Furthermore, the form directs the investigator to one of five evidence-based decisions regarding the results. Path A indicates two or more predictive concerns, Path B includes one predictive concern, Path C includes nonpredictive concerns, Path D includes parental difficulties in communication, and Path E includes no concerns. The PEDS interpretation form contains an algorithm to decide whether to refer, screen further, or observe the child, or counsel or reassure the parents on the results obtained [33].

Sheel et al. [20] reported that the PEDS was restandardized and revalidated in 2013 [15]. The interrater reliability was 0.95, and the test–retest reliability was 0.88 [15]. The PEDS accurately and rapidly differentiates children with developmental delay with a sensitivity of 86% and specificity of 74% [34]. The PEDS is considered one of the promising measures for use across settings in LMIC [12].

### 2.2. Strengths and Difficulties Questionnaire (SDQ)

The SDQ was developed in the United Kingdom. This screening measure evaluates mental health problems in children aged 2–17 years [16]. It comprises 25 questions under five domains: (a) emotional symptoms, (b) conduct problems, (c) hyperactivity/inattention, (d) peer-relation problems, and (e) prosocial behavior, and parents and teachers complete it [14]. This screening tool includes a 3-point rating scale: *not true*, *somewhat true*, and *certainly true*. The scoring for the SDQ comprises the total difficulty score, which is obtained by summing the scores for four scales, excluding the prosocial scale. The total score ranges from 0 to 40. The cut-off points for the SDQ scores are “normal”, “borderline”, and “abnormal” [16].

Sheel at el. [20] reported that Kresten et al. [35] found the SDQ has sound psychometric properties. The screening measure internal consistency is 0.73, and the test–retest reliability is 0.62. The discriminative and convergent validity are 0.80, and 0.50, respectively, and the specificity and sensitivity are above 70% [35].

## 3. Translation of PEDS and SDQ to Hindi

The current study utilized the guidelines recommended by Guillemin et al. [36]. These guidelines recommend translation, back translation, committee meetings, expert review of this translation and back translation, and pilot-testing interviews ensuring cultural acceptability and face validity of the tool.

Expert and professional judgments are often used to assess measures’ content and face validity. Face validity is the extent to which a measure appears to the user to reflect what it is intended to measure [37]. Although face validity is not a true measure of validity, it is essential for obtaining participant cooperation and engagement, so it is typically considered during preliminary checks of questionnaires [38]. However, what experts may consider suitable face validity may not be appropriate for service users [39]. Therefore, to ensure face validity, it is vital to conduct pilot testing interviews with service users to determine any discrepancies and difficulties between the original English and translated Hindi questionnaires.

### 3.1. Translation

Guillemin et al. [36] stated that translations are of higher quality when undertaken by at least two translators. Therefore, two independent translators conducted the forward translation process. The PEDS and SDQ were translated into the target language, Hindi. The translators were native to the target language and culture. They were aware of the objective underlying the material to be adapted and the concepts involved, to provide more restitution of the intended measurement [36]. To ensure quality, the two translators helped identify discrepancies in translation interpretations [40].

### 3.2. Back Translation

Back translation helps to improve the quality of the final version of the instrument and compromises one or multiple translators [36]. This method helps to highlight translation errors that may have occurred in the forward translation and would impact the study’s validity [36]. Two additional translators who were also native to the target language and culture conducted the back translation for the two forms (PEDS and SDQ). Back translation aimed to determine any discrepancy between the original and translated forms and help improve the overall quality of the final version of the translated questionnaire. A similar process was followed by Juneja et al. [41], where the authors translated and back-translated the Ages and Stages Questionnaire to screen children with DD in India.

The forward-back translations used bilingual translators. The translators’ first language was Hindi, they all completed a degree in English or Hindi and worked as language translators of content and video scripts for Indian government schools and organizations.

### 3.3. Committee Meeting

Once the translation and back translations were completed, one of the authors (HS) chaired a meeting with the four translators to proofread the translated questionnaires and compare the preliminary translation with the original English questionnaires. There were three questions in the PEDS and six questions in the SDQ that had discrepancies in the forward–back translation process. During the meeting, the author and the translators resolved the questions with more culturally appropriate words and phrases. Thus, the panel (author and the translators) agreed on the screening measure translation quality.

### 3.4. Expert Review

Two experts in the field of DD and SEL, with experience in working with young children, fluent in both English and Hindi languages, and registered with the Rehabilitation Council of India (RCI), reviewed the original and final versions of the PEDS and SDQ in the domains of target language and culture [42]. The experts reviewed the measures, agreed with the translation, and found no discrepancy or difficulty between the English and Hindi translation, ensuring face validity and cultural acceptability of the questionnaires. The final version of the instrument ensured semantic, idiomatic, experiential, and conceptual equivalences before pilot testing was conducted, which is essential as reported in Dubay and Watson [29] and Guillemin et al. [36] studies.

### 3.5. Pilot Testing Interviews

Brooks et al. [43] defined pilot testing interviews as “an initial small-scale implementation that is used to prove the viability of the project idea” (p. 52). In the current study, pilot testing interviews included evaluating whether respondents understood the meaning of items, identified rarely used phrases in the local context, and determined disparity in sentence structure between the original and translated forms [44].

The pilot study was approved by the Human Research Ethics Committee of James Cook University (HREC number: H8285). The Participant Information Sheet, detailing the study and the type of information required, was provided to participants through Qualtrics. Qualtrics is a software tool that is used to conduct survey research and evaluations. The platform is fast, easy to use, and can store large volumes of data at any given time [45]. Once the participants read the information and consented to participate in the study, they were presented with the two measures (PEDS and SDQ (English and Hindi versions)). The participants completed the questionnaires, and each participated in an online interview to check their interpretation and understanding of the screening measures. The total time to complete the form and answer the interview questions was 30 min.

#### 3.5.1. Participants

A group of 55 participants were recruited to demonstrate their interpretation and understanding of the original and translated questionnaires for the pilot test. Through purposive sampling, 21 parents and 34 teachers of children aged 4–8 years were recruited from various socioeconomic backgrounds in Chandigarh and the National Capital Region, India. Inclusion criteria for participants were parents of children aged 4–8 years who were citizens of India and could read, write, and speak at least at the Primary 6 level in either English or Hindi. Inclusion criteria for teachers were teaching children aged 4–8 years who were citizens of India and could read, write, and speak at least at the Primary 6 level in either English or Hindi. Exclusion criterion was parents whose child was not currently attending school. The demographic characteristics of the parent participants are presented in Table 1.

For the parent report group, the children’s mean age was 5.9 years (SD = 1.44, range = 4–8 years), and the majority (*n* = 13, 62%) were female. Most of the children (*n* = 20, 95%) displayed Typical Development (TD). However, a few parents indicated that their child displayed speech and hearing problems, low attention span, or behavioral issues across the two screening measures.

A total of 34 teachers filled out the SDQ questionnaire regarding a specific child they were teaching. The child reference group had a mean age of 6.29 years (SD = 1.29, range = 4–8 years), and the majority (*n* = 27, 79%) were male. Most of the children (*n* = 30, 88%) displayed TD. Only four children had DD, such as speech and hearing impairment and mild to severe autism spectrum disorder.

#### 3.5.2. Procedure

The sample size for the current study was adequate for a pilot study since studies have concluded that data must be collected with purposive sampling until saturation is reached [46,47]. Parents were recruited through schools and social media tools by sharing links of the questionnaire with groups and requesting parents and teachers of children in the age range of 4–8 years to fill the questionnaires and participate in an online interview using Qualtrics.

The pilot study was conducted using Qualtrics, where participants filled out the original and translated questionnaires and participated in an online structured interview. Fully structured interviews require the questions, probes, and responses necessary to be standardized where no practitioner-based inquiries are allowed [48]. In the Qualtrics structured interviews, the researcher asked the following questions regarding the original and translated questionnaire: Was there any difficulty in understanding the English and Hindi questions? Did you find the questions upsetting and offensive? Did you find any questions confusing to understand? And did you find any discrepancy between the original English and translated Hindi questions presented to you? Participants responded with either “Yes” or “No”. If they answered yes to any question, the researcher sought further clarification on those specific questions.

#### 3.5.3. Results

Parents’ and teachers’ responses were collated and summarized. Ninety-five percent of the participants agreed that there was no discrepancy between the original English and translated Hindi Questionnaire for PEDS and SDQ. However, two teachers were unclear about the intent and context of the English SDQ questions like “Constantly fidgeting or squirming” and “Gets on better with adults than with other children”. The researcher (HS) took note of the queries and provided clarifications on those specific questions to the participants through a telephone conversation. The questions were not altered when further administered since the concerns were regarding the intent of the original questionnaire (English) and not the translation quality of the questionnaire from English to Hindi.

## 4. Psychometric Evaluation of the Translated Measures

Once the forward–back translation of the screening measures was completed, which found the PEDS and SDQ to have adequate face validity for the original and translated version of the measures, the second part of the study aimed to psychometrically evaluate the PEDS and SDQ to ensure the measures are suitable for use with Indian children. The study aimed to explore both the reliability (internal consistency and test–retest) and validity (known group validity) of the two measures.

*Reliability* is “the consistency of scores across instances of testing procedures” ([49], p. 34). *Internal consistency* is the extent of agreement between different items within a test during single administration. In contrast, *test–retest reliability* is the consistency of scores obtained by administration of the same test on separate occasions [49]. Cronbach’s alpha of >0.70 for internal consistency [50] and a Pearson correlation of >0.70 for test–retest reliability [51] are considered acceptable for demonstrating reliability. For health status measures intended for long-term use, the recommended test–retest interval is one to two weeks [52].

*Validity* refers “to the degree to which evidence and theory support the interpretation of test scores for proposed use of tests” ([49], p. 11). Based on the results of pilot testing, the current study assumed that the English and Hindi versions were equivalent. So, the data collected for psychometric evaluation used either language version, as selected by the participants.

### 4.1. Reliability: Internal Consistency

#### 4.1.1. Research Setting

The study was conducted in India, which follows the British structure in the education system with kindergarten for 4–6 years of age, primary school for grades 1–5 (children aged 6–11 years), and middle school for grades 6–8 (children aged 11–14 years [53]). The schools were categorized as private, government, and primary schools run with the municipal cooperation of the cities in which they exist [54].

Participants were recruited from private inclusive schools in rural and urban areas of Chandigarh, Himachal Pradesh, Punjab, Haryana, and the National Capital Region, India. Inclusive schools cater to children with TD and DD. *Children were categorized as DD based on their school records; clinicians assess children in government hospitals using standardized tests and present their reports to the school for admission*. Furthermore, all the states and union territories are located in North India, where people are fluent in Hindi, English, and the states’ regional languages [55].

#### 4.1.2. Participants

For this type of research, Bujang and Adnan [56] recommend a sample size of 300 children. Considering a potential attrition rate of 20% [57], 720 (360 parents and 360 teachers) participants of children with TD and additional 240 participants (120 parents and 120 teachers) of children with DD were required to be recruited for research.

For the current study, participants comprised a convenient sample of parents and teachers of 466 children: 454 with TD and 61 with DD. Data for 47 children with TD and 2 with DD were excluded due to missing data and/or because the participants did not meet the inclusion criteria. Therefore, the final sample consisted of parents and teachers of 407 children with TD and 59 children with DD. In addition, a 25-item SDQ (teacher form) was completed by 138 teachers of the children with TD and DD. Parents were asked if they could be contacted to participate in a follow-up study (either the test–retest study or the known-group validity study). If they agreed, they provided their phone number and email address.

The inclusion and exclusion criteria for the participants were the same as for the pilot study. Demographic characteristics of the participants are presented in Table 2.

#### 4.1.3. Procedure

The study received approval from the Human Research Ethics Committee (H8285) to administer the screening questionnaires to parents and teachers of children aged 4 to 8 years. Data collection was conducted online using Qualtrics from all-inclusive schools in India.

#### 4.1.4. Results

Cronbach’s alpha [58] was used to determine the internal consistency for the PEDS, and the five subscales and Total Difficulties score of the SDQ [15,16]. Reliability was considered acceptable if >0.70 [50].

The PEDS internal consistency was acceptable (0.83 for parents of children with TD and 0.73 for parents of children with DD). The internal consistency reliability for most of the SDQ subscales, specifically Peer Problem for parents and teachers of children with TD and DD, was below standard (Table 3).

### 4.2. Reliability: Test–Retest

#### 4.2.1. Participants

To determine the sample size for test–retest reliability with an interval of 2 weeks across all questionnaires [52], a power analysis was conducted using G*power to test correlation, a medium effect size of (d = 0.3) and an alpha of 0.05. Based on the assumptions, the minimum sample size required was 84 [59].

A test–retest procedure evaluated the consistency of scores for the two questionnaires (PEDS and SDQ). In the current study, 368 parents of the children with TD and DD provided consent to be contacted for the retest, which involved completing the measures online again after two weeks. However, only 48 parents (42 parents of children with TD and 6 parents of children with DD) completed the questionnaires for the retest. Demographic characteristics of these participants are presented in Table 2.

#### 4.2.2. Results

Test–retest reliability was calculated using Pearson Product Moment correlation to estimate the level of agreement between the test and the retest [16,60,61,62,63]. Generally, reliability is considered acceptable if >0.70 [51]. More detailed guidelines recommend that <0.40 is considered poor, 0.40 to 0.59 is fair, 0.60 to 0.74 is good, and >0.74 is excellent [64].

Table 4 provides the test–retest reliability of the PEDS and the subscales of the parent report on the SDQ. The retest occurred two weeks after the initial test. Results for the SDQ subscales were variable, with fair to good reliability for emotional symptoms, hyperactivity, prosocial behavior, and the total difficulty scale. Test–retest reliability for the two groups on the PEDS was poor.

### 4.3. Validity: Construct-Related

#### 4.3.1. Participants

Thirty-eight participants from the urban population were recruited to test known-group validity. The first group comprised 24 TD participants from the higher and lower PEDS quartile, and the second group comprised 14 participants with DD. Groups were formed using a random number generator but were matched on gender and age. Demographic characteristics of the participants are presented in Table 2.

#### 4.3.2. Procedure

A sample of children with TD and DD were assessed using established diagnostic measures to establish known groups. Face-to-face assessment was conducted on the school premises or in a place convenient to the participants. Children were administered the original English language Kaufman Brief Intelligence Test—Second edition (KBIT-II; ref. [65]), and parents and teachers of the children were interviewed to complete the parent/caregiver and teacher forms of the Vineland Adaptive Behavior Scale—Third edition (VABS-3; ref. [66]). The KBIT-II and VABS-3 results were used to validate group membership, TD or DD. Parents were present during the KBIT-II assessment of their child.

#### 4.3.3. Results

To confirm group membership, three *t*-tests were conducted to examine the differences between the two groups (TD and DD) on each of the three criterion measures: the KBIT-II, VABS-3 parent form, and VABS-3 teacher form. Chi-square analysis was used to assess the construct validity of PEDS in distinguishing between the known groups (TD and DD).

On the KBIT-II, the difference between the TD group (mean = 99.54, SD = 15.34, range = 68–130) and the DD group (mean = 83.86, SD = 14.84, range = 67–109) was statistically significant (*t*(36) = 3.07, *p* = 0.004). The results from the clinical assessment found that, on average, the TD group scored higher than the DD group on the measure of general intelligence.

On the VABS-3 parent form, the difference between the TD group (mean = 109.04, SD = 14.31, range = 87–134) and the DD group (mean = 92.43, SD = 12.62, range = 72–113) was statistically significant (*t*(36) = 3.59, *p* = 0.001). Parents reported that children with TD displayed more adaptive behavior than children with DD on the VABS-3 parent form.

On the VABS-3 teacher form, the difference between the TD group (mean = 110.17, SD = 13.21, range = 89–136) and the DD group (mean = 93.07, SD = 13.21, range = 72–117) was statistically significant (*t*(36) = 3.94, *p* = < 0.001). Teachers reported that children with TD displayed more adaptive behavior than children with DD on the VABS-3 teacher form.

The results from the KBIT-II and VABS-III comparisons supported the difference between the two groups. To assess the construct validity of the PEDS, a chi-square analysis was conducted on groups by PEDS outcome *X^2^* (4, *n* = 38) = 21.14, *p* = < 0.001. The known groups were rated as significantly different on the PEDS by their parents, with parents of children with TD reporting less concerns than parents of children with DD on PEDS. This provides support for the construct validity of the PEDS in distinguishing between children with TD and DD.

## 5. Discussion

This report described the translation and psychometric examination of the PEDS and SDQ when used to assess children with TD and DD in India. The forward–back translation procedure with the PEDS and SDQ was effective as no discrepancies were found between the original and translated Hindi version of the questionnaires.

Epstein et al.’s [67] study concluded that the forward–backward translation method aids in reducing discrepancies between the original and source document resulting in more satisfactory results. Rigorous translation provides an equivalence between the two versions of the questionnaires, ensuring that any differences detected result from the differences between the groups and not from contrasts inherent in the measurement tool used to gather the data [68]. All participants in the translation and pilot study agreed that there were no discrepancies or difficulties in the PEDS and SDQ English and Hindi language forms. This outcome gave us confidence for establishing the cultural acceptability of the questionnaires. Therefore, the results from the current study support DuBay and Watson’s [29] argument of the necessity for a rigorous approach to translation and validation of screening measures where cultural differences exist.

Psychometric evaluation of the measures indicated that the internal consistency for PEDS was acceptable. However, the internal consistency for both parent report and teacher report SDQ for children with either TD or DD was less than recommended by established guidelines in most SDQ domains. Specifically, the Peer Problem scale for parents and teachers of children with TD and DD was well below the recommended level. The test–retest reliability was poor for PEDS and generally adequate for the parent report SDQ. The use of the KBIT-II and VABS-3 (parent and teacher forms) provided objective assessment of group membership for the examination of the validity of the PEDS.

In the current study, the PEDS had a robust internal consistency, similar to the findings from studies conducted within community samples in LMICs, such as Tehran [15,69]. However, the test–retest reliability for the PEDS with TD children was poor. This contrasts with results reported by Glascoe’s [15] study and research conducted in LMIC countries, like Tehran [69], and with translated Dutch [70] and Mandarin [71] versions of the PEDS, all of which have reported acceptable test–retest reliability.

One possible reason for the poor test–retest reliability found in the current study is that it is likely that after the initial PEDS administration, most parents realized their children could perform tasks they had not thought they would be able to [72]. In support of this explanation, findings in the current study showed that parents’ concerns significantly diminished following the initial screening. Further, the lack of routine screening results in low developmental literacy in parents in LMICs compared to those in high-income countries. Therefore, the initial PEDS administration could have acted as training for parents in what to observe in their children, resulting in different responses being given on the second administration of the PEDS. As the PEDS was not administered in an interview format, parents were not able to describe and review their concerns with the interviewer. Therefore, it is plausible that they would have reflected on the PEDS items after the first administration, hence reporting less concerns in the retest [73].

The test–retest reliability for parents of children with DD was negative. However, with a small sample size of only six participants, these findings cannot be considered meaningful, leading to inaccurate results [51].

Glascoe [33] evaluated the concurrent validity of the PEDS with the Kaufman Assessment Battery for Children and the Vineland Adaptive Behavior Scale. The results indicated a moderate to high correlation between the PEDS domains and the diagnostic measures. The measures used in the current study, the KBIT-II and VABS-3, have been found to differentiate between children with TD and DD [74] and were used here to provide validation of group membership. The results from the PEDS could distinguish between the known groups of children with TD and DD, providing evidence for the concurrent validity of PEDS scores. These current results are like those reported in a South African study, where most parents of children at risk of DD had higher concerns than parents of their age-equivalent peers on the developmental milestones of behavior, school, cognitive development, and health [75].

On the SDQ, the internal consistency found in the current study was very similar to findings from other LMIC, such as the Democratic Republic of the Congo (parent and teacher; community sample; [76]), Vietnam (parent; clinical and community samples; [77]) and South Africa (caregivers; community sample; [78]), and High-Income Countries, such as Australia (parent; community sample; [79]), Germany (parent; community sample; [80]), Sweden (parent; clinical and community samples; [81]), Netherlands (parent; community samples; [82]; parent and teacher; community samples; [83]), New Zealand (parent and teacher; community samples; [84]), and the United Kingdom (parent; community samples; [85]; parent, community; [86]). However, even higher internal consistency reliability has been reported in Turkey (parent; community sample; [87]) and Germany (parent and teachers; community sample; [88]).

In the current study, only six (25%) of the internal consistency correlations where above the cut-off of 0.70. The results for the peer problem subscale were particularly poor. Multiple factors could contribute to the wide range of the results obtained. First, Van Widenfelt et al.’s [83] study found that low internal consistency for the peer problems subscale could be due to one or two items not fitting with the remaining scale and parents and teachers partially reporting existing problems, probably due to being only partly aware or willing to report concerns on this subscale. Second, the SDQ consists of only five items per subscale [82,83], and studies have reported that the low internal consistency of the SDQ subscales could be attributed to the negative items in the five items of each subscale contributing to measurement error [89]. Third, items in the peer problem subscale are strongly linked with ASD, which includes social and communication impairments, whereas the current study consisted of children with no disability and a range of developmental disabilities [82,90]. Lastly, for the DD sample, the highest educational qualification for 39% of the parents was middle school, which may have limited their ability to understand and interpret the questions.

The current findings for test–retest reliability are similar to those reported in Australia [91] and Japan [61], where the authors also provided a 2-week test–retest period. In contrast, in the original study, Goodman [16] provided a test–retest period of four to six months and reported moderate correlation coefficients. However, Goodman mentioned that the period he used for test–retest should not be considered suitable as it was too long.

It is unclear what the most suitable test–retest interval for assessing the reliability of the SDQ is. Polit [52] and Streiner et al. ([92], p. 172) recommended a two- to three-week gap for obtaining accurate reliability. In contrast, Muris et al. [82] used a period of 2 months and reported high reliability on the parent report SDQ. Studies carried out in Finland [93] and Australia [79] found similar test–retest reliability coefficients as the current study, even though the retest periods were 12 weeks and 12 months, respectively.

Systematic reviews of the SDQ’s psychometric properties have reported low test–retest reliability [35]. Specifically, test–retest reliability is generally low for the parent report group compared to that of the teacher report over time [94]. Overall, there is not a large body of research findings on the SDQ’s test–retest reliability, and more research needs to be conducted to explore the SDQ’s psychometric properties across cultures.

## 6. Limitations and Future Recommendations

Despite the rigorous translation process and the large sample recruited for the first administration of the measures, the sample sizes were small for test–retest (*n* = 48) and known-group (*n* = 38) validity testing. Furthermore, the study consisted of only children aged 4–8 years and from a few states in North India; this is not a national representation of India, which has 28 states and multiple languages. Furthermore, since the data were s collected during the COVID-19 pandemic, caution is recommended in generalizing the results for a non-pandemic environment where physical attendance at school is the norm.

Future studies could conduct face-to-face pilot testing interviews for a more detailed evaluation of the screening tools [95]. Moreover, India is multilinguistic, so the questionnaires may need to be adapted to regional languages to cater for a specific population. Studies have shown that parents of children from low-socioeconomic backgrounds, who often speak their native languages, can better report on their children when interviewed in that native language [96].

Despite this limitation, this study is the first attempt to validate the PEDS and SDQ using parents and teachers of children with TD and DD in India. This study included children from rural and urban North India, making the results generalizable beyond the usual urban-based studies. Furthermore, PEDS and SDQ were translated into Hindi, allowing schools and clinics use of the tool for screening.

## 7. Conclusions

In summary, by following a rigorous translation and pilot testing procedure, this study demonstrated the face validity and cultural accessibility of the PEDS and SDQ for the Indian population. These positive results also raise the possibility of these measures being suitable for use in other LMICs. While the findings for reliability were mixed, there was evidence for the construct validity of the PEDS. The issues negatively impacting some of the SDQ reliability coefficients were most likely a function of the nature of the items and the lack of parental awareness of developmental phases in childhood.

It is vital to consider the critical construct and language issues when adapting measures standardized for Western cultures for use in the Asian context [1]. However, this study demonstrates that these issues are not insurmountable. The results also suggest that the assessment process can be a way of educating parents on best practices to promote their child’s development. The assessment process can be expanded to include information about vigilant monitoring and childrearing, as well as provide parent training sessions [33].

## Figures and Tables

**Table 1:** Demographic Characteristics of the Pilot Study Participants (Parents).

Demographic Characteristics	*n*	%
Parent		
Mother	19	90
Father	2	10
Highest Educational Qualification		
Certificate/Diploma	2	10
Undergraduate Degree	1	4
Postgraduate Degree	18	86
Yearly Household Income		
<75,000 INR *	4	19
INR 0.75–1.5 lakhs	1	5
INR 1.6–3 lakhs	1	5
INR 3.1–5 lakhs	4	19
INR 5.1–10 lakhs	3	14
>10 lakhs	8	38

Note. * A lakh in Indian rupees is equivalent to one thousand US dollars.

**Table 2:** Demographic Characteristics of Participants (Parents) for the Psychometric Evaluation Studies.

Demographic Characteristics	Reliability (Internal Consistency) (*n* = 466)		Reliability (Test–Retest) (*n* = 48)		Validity (Known Group) (*n* = 38)
	TD	DD	TD	DD	TD and DD
Gender of the Child					
Males	259 (64%)	43 (73%)	27 (64%)	5 (83%)	22 (58%)
Females	148 (36%)	16 (27%)	15 (36%)	1 (17%)	16 (42%)
Parent					
Mother	276 (68%)	41 (69%)	33 (79%)	3 (50%)	27 (71%)
Father	131 (32%)	18 (31%)	9 (21%)	3 (50%)	11 (29%)
Highest Educational Level					
Middle school	14 (3%)	23 (39%)	3 (7%)	0 (0%)	7 (18%)
High school	35 (9%)	4 (8%)	1 (2%)	0 (0%)	1 (3%)
Diploma	21 (5%)	5 (8%)	1 (2%)	0 (0%)	2 (5%)
Undergraduate degree	118 (29%)	15 (25%)	12 (30%)	5 (83%)	12 (31%)
Postgraduate degree	219 (54%)	12 (20%)	25 (59%)	1 (17%)	16 (42%)
Yearly household income					
<75 k	67 (16%)	28 (47%)	7 (17%)	1 (17%)	11 (29%)
75 k–1.5 Lac	55 (14%)	6 (10%)	5 (12%)	0 (0%)	8 (21%)
1.6–3 Lac	42 (10%)	9 (15%)	2 (5%)	1 (17%)	7 (18%)
3.1–5 Lac	90 (22%)	4 (8%)	9 (21%)	0 (0%)	4 (10%)
5.1–10 Lac	88 (22%)	11 (19%)	10 (24%)	4 (66%)	4 (66%)
>10.1 Lac	65 (16%)	1 (1%)	9 (21%)	0 (0%)	2 (5%)

Note. TD = Typical Development, DD = Developmental Disability.

**Table 3:** Internal Consistency of the SDQ with Parents and Teachers of Children with TD and DD.

SDQ Subscales	TD		DD	
	Parent	Teacher	Parent	Teacher
Total Difficulties	0.63	0.78	0.75	0.76
Emotional Symptoms	0.61	0.82	0.56	0.57
Conduct Problem	0.49	0.56	0.61	0.63
Hyperactivity	0.53	0.71	0.62	0.62
Peer Problem	0.30	0.40	*	*
Prosocial Behavior	0.61	0.78	0.63	0.63

Note. * represents a negative internal consistency tabulated using Cronbach’s Alpha.

**Table 4:** Test–Retest Reliability for PEDS and Parent report SDQ.

Measure	Test–Retest Correlation	
	TD	DD
PEDS	0.45 *	−0.29
SDQ Subscales		
Total Difficulties	0.73 *	0.81 *
Emotional Symptoms	0.72 *	0.89 *
Conduct Problem	0.52 *	0.57
Hyperactivity	0.64 *	0.80
Peer Problem	0.52 *	0.45
Prosocial Behavior	0.66 *	0.63

Note. * *p* < 0.01.

## Data Availability

The data are available at https://research.jcu.edu.au/data/default/rdmp/home (accessed on 12 December 2023)—https://research.jcu.edu.au/data/published/b19b543076d211ed9b9a51fb9846a249/ (accessed on 12 December 2023).
